# Moisture Distribution and Ice Front Identification in Freezing Soil Using an Optimized Circular Capacitance Sensor

**DOI:** 10.3390/s24227392

**Published:** 2024-11-20

**Authors:** Xing Hu, Qiao Dong, Bin Shi, Kang Yao, Xueqin Chen, Xin Yuan

**Affiliations:** 1 Department of Roadway Engineering, Southeast University, Nanjing 211189, China; 220223148@seu.edu.cn (X.H.); sb0523@seu.edu.cn (B.S.); kangyao@seu.edu.cn (K.Y.); 213223365@seu.edu.cn (X.Y.); 2 Department of Civil Engineering, Nanjing University of Science and Technology, Nanjing 210094, China; xueqinchen2008@126.com

**Keywords:** ice front, moisture distribution, frozen soil, electrical capacitance tomography, three-dimensional reconstruction

## Abstract

As the interface between frozen and unfrozen soil, the ice front is not only a spatial location concept, but also a potentially dangerous interface where the mechanical properties of soil could change abruptly. Accurately identifying its spatial position is essential for the safe and efficient execution of large-scale frozen soil engineering projects. Electrical capacitance tomography (ECT) is a promising method for the visualization of frozen soil due to its non-invasive nature, low cast, and rapid response. This paper presents the design and optimization of a mobile circular capacitance sensor (MCCS). The MCCS was used to measure frozen soil samples along the depth direction to obtain moisture distribution and three-dimensional images of the ice front. Finally, the experimental results were compared with the simulation results from COMSOL Multiphysics to analyze the deviations. It was found that the fuzzy optimization design based on multi-criteria orthogonal experiments makes the MCCS meet various performance requirements. The average permittivity distribution was proposed to reflect moisture distribution along the depth direction and showed good correlation. Three-dimensional reconstructed images could provide the precise position of the ice front. The simulation results indicate that the MCCS has a low deviation margin in identifying the position of the ice front.

## 1. Introduction

Seasonal frozen areas are widely distributed, covering 53% of the world’s land surface [1,2,3]. The frost heave and thaw settlement of frozen soil caused by seasonal temperature fluctuations endanger the safety of various engineering facilities in seasonally frozen areas. Current studies have found that phase transitions and the migration of internal moisture during the freeze–thaw process, along with the movement of the freezing–melting boundary (the ice front), are the main triggers of engineering failures in seasonally frozen regions [4]. Thus, accurately identifying the location of the ice front in frozen soil and understanding the internal moisture distribution is crucial for disaster prevention and reduction in seasonally frozen areas.

Currently, various measurement techniques, including ground-penetrating radar [5], CT techniques [6,7], the electric resistance measuring method [8], and portable nuclear magnetic resonance detectors [9], can be used to measure the composition distribution of frozen soil and monitor the movement of the freezing boundary. Bittelli et al. used dielectric spectroscopy to estimate the ice content in frozen porous media and derived the volume fractions of different phases in the medium based on the volume and dielectric constant of each phase [10]. Kunio et al. simultaneously measured the liquid water content and relative permittivity of various unsaturated soils at above-zero and subzero temperatures by using pulsed nuclear magnetic resonance (NMR) and time-domain reflectometry (TDR) [11]. Wen et al. used an NMR technique and thermal resistor temperature probe to characterize unfrozen water content and soil matric potential [12]. Generally, it is hard to achieve real-time and non-invasive measurements on the freezing interface of the frozen soil. Given the complexity and nature of the issue, finding a reliable approach to measure the composition distribution and monitor the movement of the freezing interface is still a critical challenge.

Electrical capacitance tomography (ECT) is one of the techniques employed in industrial process tomography (IPT), which can be used to evaluate the spatial distribution of permittivity based on current and voltage conditions along the boundaries of a specified region [13]. The measurement principle of ECT is based on the fact that the capacitance of a capacitor is a function of the permittivity (ε) of the medium between the electrode plates over the entire sensing space [14,15]. To inversely calculate the relative permittivity, the measured capacitance values are processed using an appropriate image reconstruction algorithm [16,17]. Electrical capacitance tomography has the advantages of a rapid response, low cost, non-intrusiveness, and robustness in harsh environments [18,19]. Compared to previous moisture detection methods in frozen soil, the ECT has great potential for evaluating moisture distribution and identifying ice fronts inside frozen soil due to its high sensitivity to water [20,21,22].

The objective of this paper is to design and optimize a mobile circular capacitance sensor (MCCS). This is employed in frozen soil testing to obtain the moisture distribution and the position of the ice front. Finally, the MCCS’s measurement indexes are validated based on the finite element simulation results. The research is divided into four goals: (a) to optimize MCCS’s structural parameters based on fuzzy optimization design; (b) to evaluate the moisture distribution along the height of the specimen based on the two proposed indexes; (c) to reconstruct the 3D ice front image via trilinear interpolation; (d) to obtain the simulated unfrozen moisture content to verify the MCCS’s evaluation index.

## 2. ECT Principle

The basic principle of the CCS used in this study is based on the fringe effect [23]. One of the electrodes of the CCS is applied to an AC or DC voltage as the excitation electrode, generating an electric field between the other electrodes, which are kept at zero potential and serve as the detectors. The capacitance between the excitation and the detection electrodes varies with the permittivity changes due to the different material distributions inside the sensor [24].

**Figure 1:**
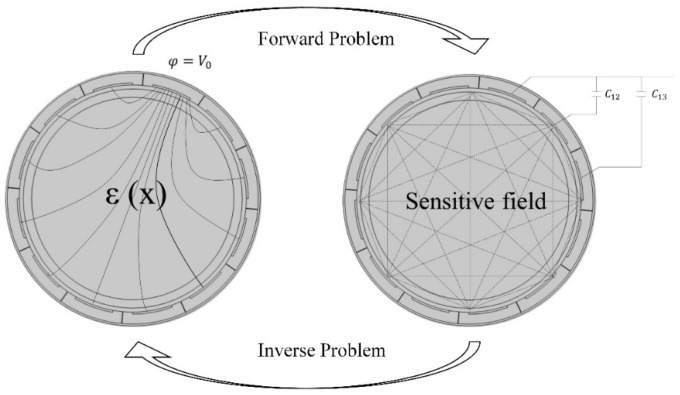
Schematic diagram of ECT’s forward and inverse problems [17].

The essence of the ECT imaging problem is the need to solve two major mathematical problems, forward and inverse [16], as shown in Figure 1. The forward problem can be explained as follows: the sensor structure, medium distribution, and electrode measurement strategy are set up to solve the electrical field and potential distribution in the measurement field and obtain the sensitivity matrix. The inverse problem inversely calculates the medium distribution using image reconstruction algorithms based on the measured capacitance data and forward solution. Thus, the key to the forward problem is to solve the capacitance value of the medium distribution based on the set condition.

The following Maxwell equations represent the macroscopic electromagnetic field in the array capacitive sensor [25]:

(1) $$\left\{\begin{array}{c} \nabla \times H=J+\frac{\partial D}{\partial t}\\ \nabla \times E=-\frac{\partial B}{\partial t}\\ \nabla \cdot B=0\\ \nabla \cdot D=\rho \end{array}\right.$$

where H is the magnetic field intensity, E is the electric field intensity, B is the magnetic induction intensity, D is the electric flux density, J is the current density, and ρ is the charge density.

The operating frequency of the excitation electrode is in the low-frequency range, which corresponds to the electrostatic field condition, and the basic equation of the electrostatic field is obtained:

(2) $$\left\{\begin{array}{c} D=\varepsilon E\\ \nabla \cdot D=0\\ E=-\nabla \phi \\ \nabla \times E=0 \end{array}\right.$$

where ε is the permittivity and ϕ is the electric potential.

According to the above equations, the mathematical model of the ECT system is derived as the Poisson equation:

(3) $$\nabla \cdot \left(\varepsilon \cdot \nabla \phi \right)=0$$

Based on the Gauss formula, the capacitance between the i and j electrode pairs of the forward problem is as follows:

(4) $$C_{i,j}=\frac{Q}{V}=\frac{1}{V}\iint_{\Gamma }\varepsilon \left(x,y\right)\nabla \Phi \left(x,y\right)d\Gamma$$

where $\varepsilon \left(x,y\right)$ is the permittivity distribution in the sensing field; *V* is the potential difference between the two electrodes forming the capacitance; $\Phi \left(x,y\right)$ is the potential distribution; *Γ* is the electrode surface. Equation (4) can be written in the following form:

(5) $$C=\iint_{\Gamma }\varepsilon \left(x,y\right)\cdot S\left(x,y,\varepsilon \left(x,y\right)\right)dxdy$$

where $S\left(x,y,\varepsilon \left(x,y\right)\right)$ is the sensitivity matrix. The sensitivity matrix reflects the sensitivity of the capacitance values between electrodes to changes in the permittivity in the sensing field. The permittivity distribution $\varepsilon \left(x,y\right)$ is inverted by the capacitance value and the sensitivity matrix using an image reconstruction algorithm to characterize the moisture distribution of frozen soil.

## 3. Material and Methods

### 3.1. Fuzzy Satisfaction Evaluation Method

The evaluation indexes for circular capacitive sensors are typically associated with the capacitance measurements and sensitivity field distribution. This paper chooses capacitive dynamic range and the uniformity of sensitivity distribution as the two indicators for assessing MCCS performance, both of which can be calculated using COMSOL Multiphysics. The capacitive dynamic range ($R_{c}$) is defined as the ratio of the maximum capacitance ($C_{max}$) measured under full-field conditions to the minimum capacitance ($C_{min}$) measured in a null field, as indicated in Equation (6). The capacitive dynamic range should not be excessively high to minimize noise interference.

(6) $$R_{c}=\frac{C_{max}}{C_{min}}$$

Non-uniform sensitivity can severely impact image quality. In the MCCS system, the arrangement of electrode plates is symmetrical, and the sensitivity field distribution ($S_{i,j}$) between any two plates remains consistent upon rotation. Therefore, only half of the number of electrode plates needs to be considered in the sensitivity distribution, with the calculation formula as shown in Equation (7).

(7) $$\left\{\begin{array}{c} S_{i,j}^{avg}=\frac{1}{n}\sum_{e=1}^{n}S_{i,j}(k)\\ S_{i,j}^{dev}={\left\{\frac{1}{n-1}\sum_{e=1}^{n}{\left[S_{i,j}\left(k\right)-S_{i,j}^{avg}\right]}^{2}\right\}}^{1/2}\\ D=\frac{2}{P}\left(\sum_{i=1}\sum_{j=2}^{\frac{2}{P}+1}\left|\frac{S_{i,j}^{dev}}{S_{i,j}^{avg}}\right|\right) \end{array}\right.$$

where $S_{i,j}^{avg}$ is the average sensitivity; $S_{i,j}^{dev}$ is the standard deviation of n micro-elements in the measured area; D is defined as the sensitivity distribution coefficient; P is the number of the electrode plates. A smaller value of D indicates a more uniform distribution of sensitivity.

Due to the numerous optimization indicators involved in ECT sensors, there may be conflicts among these indicators. When one indicator is optimal, the others are not necessarily optimal. To alleviate the conflicts between indicators, a reasonable combination of multiple indicators is necessary, followed by an experimental evaluation of the influencing factors. This paper employs the multi-index orthogonal experimental fuzzy analysis method proposed by Ji [26], establishing satisfaction levels regarding the indicators and their corresponding membership functions based on the concepts of fuzzy mathematics. The satisfaction function is defined to characterize the degree of acceptance, utilizing the existing normal distribution function to create the satisfaction function for evaluation indicators. These can be categorized into large-type, small-type, and intermediate-type indicators, with each influencing the overall evaluation results in distinct directions. Both $R_{c}$ and D are small-type indicators, and Equation (8) is used for calculation:

(8) $$\mu_{A_{j}}\left(x_{i,j}\right)=\left\{\begin{array}{cc} 1, & x_{i,j}<a\\ e^{-k{\left(x_{i,j}-a\right)}^{2}}, & x_{i,j}\geq 0 \end{array}\right.$$

where k relates the index value obtained from the experiment and the satisfaction level. In this paper, k is determined using an empirical method. In order to enhance the rationality and effectiveness of the comprehensive satisfaction function for the indicators, this paper employs a weighted arithmetic mean approach to develop the comprehensive satisfaction function, as shown in Equation (9).

(9) $$FSI\left(x_{i,j}\right)=\frac{1}{2}e^{-0.000756x_{i,1}^{2}}+\frac{1}{2}e^{-0.00582x_{i,2}^{2}}$$

where FSI is defined as the fuzzy satisfaction index.

### 3.2. Specimen Preparation and Test Method

#### 3.2.1. Material and Sample Preparation

**Figure 2:**
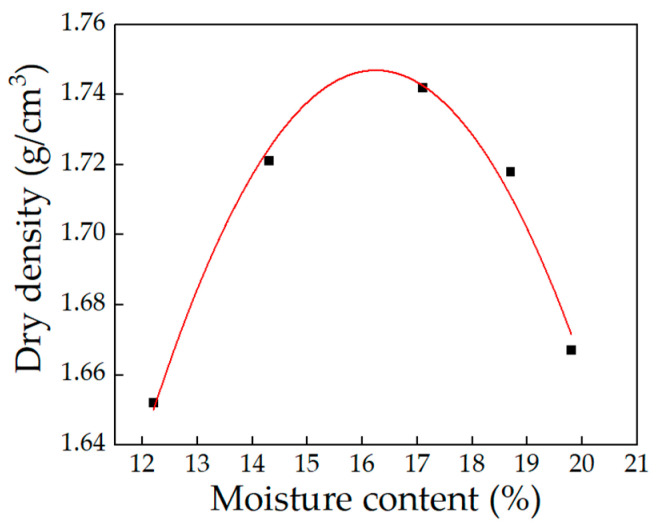
Compaction curve of loess.

The loess used was sourced from a subgrade construction project in a seasonally frozen region of China. The compaction testing was conducted according to ASTM D1557 [27]. Figure 2 presents the results of the compaction test. The maximum dry density is 1.746 g/cm^3^, with an optimal moisture content of 16.28%. Based on the optimal moisture content, the initial moisture content of the samples was set at 10%, 15%, and 20%. The prepared soil sample measured 10 cm in diameter and 16 cm in height. After preparation, the surface of the sample was wrapped with waterproof and insulating layers, made of polyethylene and polyester foam, to prevent moisture and heat exchange between the sample and its surroundings. The samples were subjected to a constant temperature of +2 °C for 24 h prior to the initiation of freezing.

#### 3.2.2. Testing Methods

**Figure 3:**
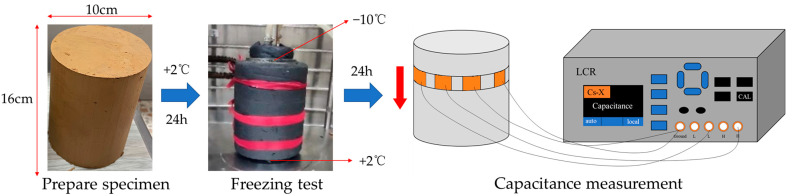
The diagram of the testing procedure.

After the sample was prepared, capacitance values were collected every 1 cm along the depth using optimized MCCS, and these values were regarded as the full field. The control samples were fully dried, and capacitance values were also collected to represent the empty field. A multifunctional freeze–thaw testing machine was used for unidirectional freezing of the soil samples. The top temperature was set to −10 °C and the bottom to 2 °C, simulating unidirectional freezing in natural soil conditions. After freezing for 24 h, the sample was removed, and measurements were taken along the depth using MCCS, with the results considered as the measured field. After capacitance testing, the sample could be sliced at 1 cm intervals along the height, and the moisture content of each slice could be measured using the oven-drying method. For capacitance measurement, a high-sensitivity ECT system based on a commercial precision LCR meter (VICTOR 4090A) (Shenzhen Yisheng Victory Technology Co., Ltd., Shen Zhen, China) was employed. The excitation voltage was set at 2V while the frequency of data acquisition was 100 kHz. Figure 3 displays the whole experimental procedure.

#### 3.2.3. Characterization Parameters Based on Capacitance Data

The sum of capacitance values ($C_{s}$) can be used to visually represent the moisture content across different layers of the frozen soil sample. However, the total capacitance value does not reflect the distribution of dielectric material across the cross-section, leading to considerable error. The average dielectric constant distribution ($\varepsilon_{m}$) is introduced as an indicator to evaluate moisture migration in frozen soil due to the substantial difference between the dielectric constants of frozen and unfrozen soil. Using the sensitivity matrix derived from prior simulations and the Landweber algorithm, the normalized dielectric constant distribution of a specific cross-section of emulsified asphalt can be calculated.

(10) $$\varepsilon_{m}=\frac{1}{n}\sum_{i=1}^{n}\varepsilon_{i}$$

where $\varepsilon_{i}$ represents the normalized dielectric constant of the *i*-th pixel.

### 3.3. Finite Element Method

Since MCCS primarily functions to detect the ice front in frozen soil, a two-dimensional thermal–hydraulic coupling model was established in COMSOL Multiphysics.

Step 1. Derivation of the mathematical model. According to Fourier’s law, latent heat from phase changes is treated as a heat source, and the differential equation for thermal conduction in frozen soil is given by Equation (11) [28,29,30].

(11) $$\rho C\left(\theta \right)\frac{\partial T}{\partial t}=\lambda \left(\theta \right)\nabla^{2}T+L\cdot \rho_{i}\frac{\partial \theta_{i}}{\partial t}$$

where T is the transient temperature of the soil (°C); t is time (s); θ is water content (%); C(q) represents the volume heat capacity of soil (J/m^3^); $\lambda \left(\theta \right)$ is thermal conductivity (W·m^−1^ K^−1^); $\theta_{i}$ is ice content (%); ρ and $\rho_{i}$ are the density of water and ice (kg/m^3^), respectively; L is the latent heat of the phase change (kJ/kg). Using Darcy’s law and the principle of mass conservation as the foundation, a water migration control equation is established [30,31,32].

(12) $$\frac{\partial \theta_{u}}{\partial t}+\frac{\rho_{i}}{\rho_{w}}\cdot \frac{\partial \theta_{i}}{\partial t}=\nabla \left[D\left(\theta_{u}\right)\nabla \theta_{u}+k_{g}(\theta_{u})\right]$$

where $\theta_{u}$ is the volumetric content of unfrozen water (%); $k_{g}(\theta_{u})$ represents the permeability coefficient of unsaturated soil in the gravity direction (m/s); $D\left(\theta_{u}\right)$ is the water diffusion coefficient (m^2^/s). the solid–liquid ratio ($B_{i}$) is introduced to solve the hydrothermal coupling equations simultaneously [21]. The volumetric ice content of ice is expressed as follows:

(13) $$\theta_{i}=B_{i}(T)\cdot \theta_{u}$$

Step 2. Establishment of the numerical simulation model. The geometric model was constructed, and Equations (11) and (12) were transformed into the coefficient form of partial differential equations in COMSOL Multiphysics.

(14) $$\left\{\begin{array}{c} \rho C\left(\theta \right)\frac{\partial T}{\partial t}+\nabla \cdot \left(-\lambda \left(\theta \right)\nabla T\right)=L\cdot \rho_{i}\frac{\partial \theta_{i}}{\partial t}\\ \frac{\partial \theta_{u}}{\partial t}+\nabla \cdot \left(-D\left(\theta_{u}\right)\nabla \theta_{u}-k_{g}\left(\theta_{u}\right)\right)+\frac{\rho_{i}}{\rho_{w}}\cdot \frac{\partial \theta_{i}}{\partial t}=0 \end{array}\right.$$

Step 3. Numerical calculation and export of results. Boundary conditions were set to match the experimental conditions, and the calculated moisture distribution results were exported.

## 4. Results and Discussion

### 4.1. Optimization Results of MCCS

Due to the large number of parameters involved in sensor optimization and the complex influence of external factors on certain parameters, it is essential to select relatively independent parameters for structural optimization. The structural parameters of MCCS primarily include the number of electrode plates, electrode opening angle coverage, plate width, and grounding shield radius. For a problem with a factor of 4 and a level of 3, all experiments must be performed 81 times. The orthogonal design method was employed to solve this problem, reducing the number of experiments required for the MCCS to nine using a three-level orthogonal table. Table 1 presents the orthogonal design layout.

The capacitive dynamic range ($R_{c}$), sensitivity distribution coefficient (D), and fuzzy satisfaction index (FSI) were selected as optimization indexes. Orthogonal experiment results are shown in Table 2. It was found that $R_{c}$ and D cannot be optimized simultaneously, indicating that a conflict will occur when they are both chosen as optimization objectives.

The range analysis of the orthogonal test results is shown in Table 3, where $R_{R}$ represents the range of the optimization indicator for $R_{c}$, $R_{D}$ represents the range for D, and R represents the range for the FSI. A can be used to indicate the number of electrode plates, B to indicate the electrode opening angle coverage, C to indicate the plate width, and D to indicate the grounding shield radius. For example, A_1_B_2_C_3_D_4_ indicates the optimal combination, where the first set of electrode plate numbers, the second set of electrode opening angle coverages, the third set of plate widths, and the fourth set of the grounding shield radius are selected for a specific function. When the optimization indicator is $R_{c}$, range analysis shows that the order of influence of the parameters on $R_{c}$ is A > D > B > C, and the optimal result is A_2_B_1_C_3_D_3_. For the optimization indicator D, range analysis determines the influence order on D as A > D > B > C, resulting in the optimal combination A_2_B_3_C_3_D_1_. The influence order of factors in the two optimization results is the same, but the corresponding level of each factor cannot be identical. With FSI as the optimization indicator, range analysis reveals that the order of influence of the parameters on $R_{c}$ is A > D > B > C and the optimal result is A_2_B_1_C_3_D_1_.

As the optimal combination derived from the orthogonal experiment was not incorporated into the simulation test plan, a simulation test was performed on the selected parameters. The optimized parameter results are shown in Table 4. Comparing the results with those of with $R_{c}$ and D, using FSI to evaluate the structural parameters of MCCS can simultaneously obtain a low-capacitance dynamic range and a uniformly sensitive field distribution.

### 4.2. Characterization of Moisture Distribution

**Figure 4:**
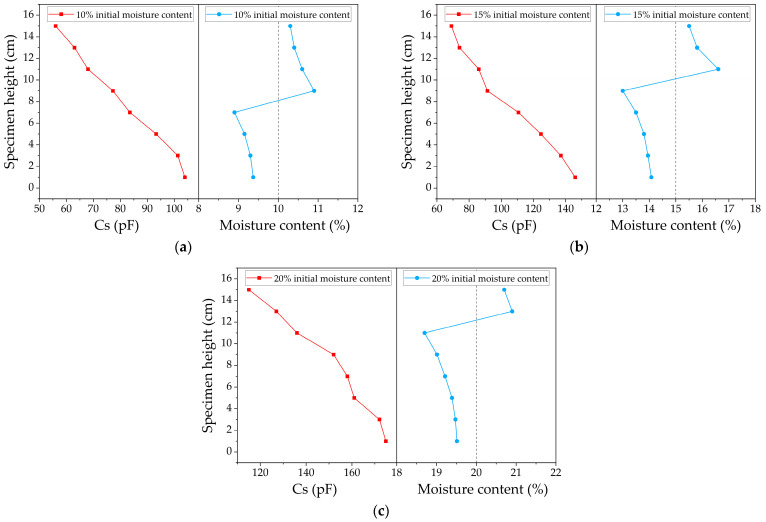
$C_{s}$ and moisture content variation in different specimens: (**a**) 10% initial moisture content; (**b**) 15% initial moisture content; (**c**) 20% initial moisture content.

**Figure 5:**
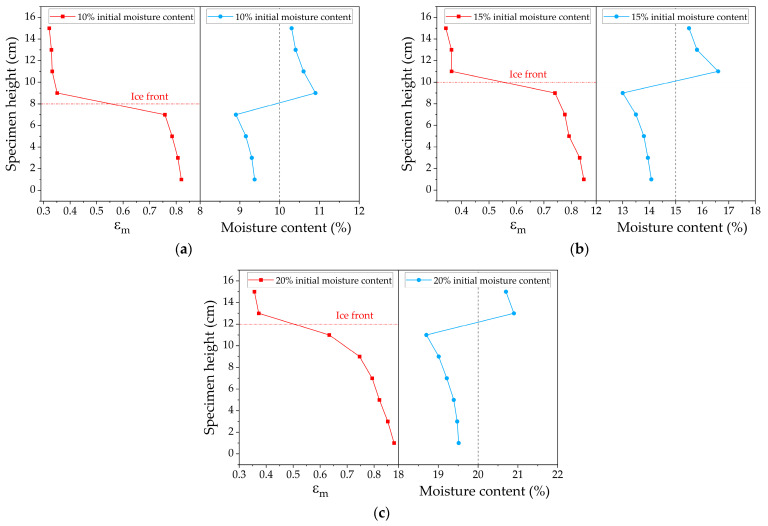
$\varepsilon_{m}$ and moisture content variation in different specimens: (**a**) 10% initial moisture content; (**b**) 15% initial moisture content; (**c**) 20% initial moisture content.

The frozen soil samples with different initial moisture contents were tested using MCCS to analyze the feasibility of using measurement indexes to assess moisture distribution. The test results are shown in Figure 4 and Figure 5. Figure 4 illustrates the distribution of the sum of capacitance values and the total moisture content along the depth of the specimen, while Figure 5 shows the average dielectric constant distribution and the total moisture content along the same depth direction. It was found that the moisture content in the unfrozen area decreased while the moisture content in the frozen area increased. As the experiment was conducted in a closed system with no external water supply, it is assumed that moisture migrated from the unfrozen area to the frozen area. As the initial moisture content increased, the slope of the curves in the figure increased, indicating more significant variations in moisture content along the height direction. Based on the increase in the order of initial moisture content, the peak moisture content positions for the 10%, 15%, and 20% samples were 8 cm, 10 cm, and 12 cm, respectively, with peak moisture content increasing by 9.5%, 11.3%, and 5.3% compared to the initial values.

As the initial moisture content increased, more water accumulated in the frozen region, and less water remained in the unfrozen region, showing a trend of increasing moisture migration. This is because, under the same cold junction temperature conditions, a higher initial moisture content in the samples meant that more time was required for freezing, providing more time for moisture migration and leading to a greater migration volume. Conversely, with a low initial moisture content in the sample, the freezing speed increased, resulting in a shorter migration time and a smaller migration volume.

It can be seen from the $C_{s}$ curves of the three specimens that there was a uniform decreasing trend from top to bottom, attributed to the much lower relative dielectric constant of ice compared to water. However, the slope variations showed no discernible pattern. The $\varepsilon_{m}$ curves also showed a monotonically decreasing trend from bottom to top, but the greatest slope change occurred at the peak moisture content, indicating the most significant change in the dielectric properties of the material, with clear ice crystal formation at this location. If the $\varepsilon_{m}$ value is less than this point, it is considered to be a frozen region, and if the $\varepsilon_{m}$ value exceeds this threshold, it is considered to be an unfrozen region. Notably, the moisture content here was measured using the oven-drying method, which accounted for the migrated and frozen water. Therefore, both curves exhibited a monotonically decreasing trend.

**Figure 6:**
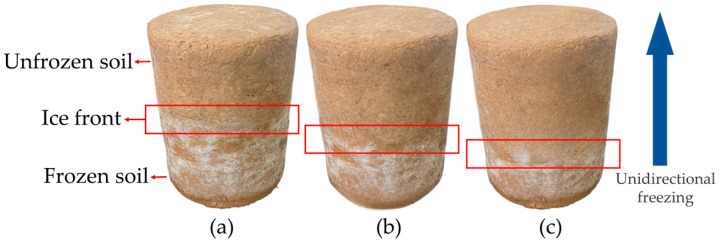
Ice fronts of different specimens: (**a**) 10% initial moisture content; (**b**) 15% initial moisture content; (**c**) 20% initial moisture content.

A clear boundary between the frozen and unfrozen zones appeared near the peak moisture content, as illustrated in Figure 6, indicating that the freezing front occurred at the moisture content peak. Due to the characteristics of the material, the point with the minimum slope in the $\varepsilon_{m}$ curve can be considered the ice front. It was found that the freezing fronts identified by the two methods were consistent, which preliminarily verified the accuracy of using $\varepsilon_{m}$ to assess moisture distribution. Since the measurement step was 1 cm, the freezing front obtained using this indicator could only be taken as the intermediate value between two measured heights; an error analysis will be performed in the following finite element simulations.

### 4.3. Three-Dimensional Imaging of Ice Fronts

**Figure 7:**
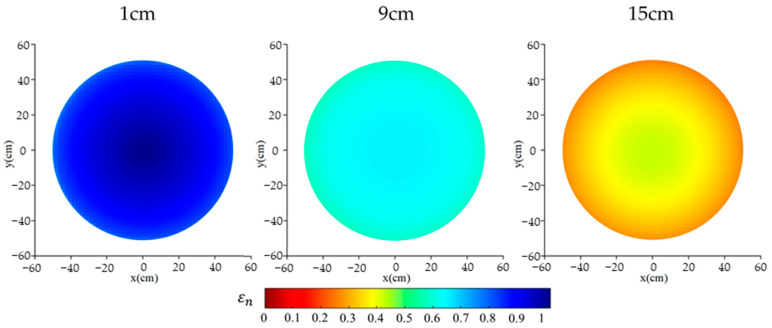
2D image of the relative permittivity distribution at different specimen heights with 10% initial moisture content.

**Figure 8:**
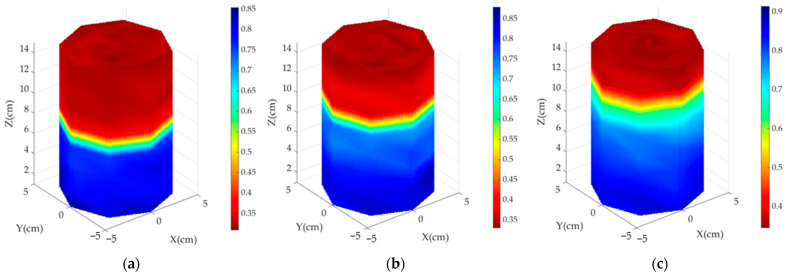
Three-dimensional interpolated cloud plot of relative permittivity: (**a**) 10% initial moisture content; (**b**) 15% initial moisture content; (**c**) 20% initial moisture content.

Before calculating the average dielectric constant distribution, a 2D image of the material distribution within the measured cross-section can be obtained. As shown in Figure 7, the dielectric constant distribution matrix was converted into a 2D image. Due to the presence of waterproof and insulation layers on all sides, the moisture distribution in the radial direction of the specimen showed little variation. Using trilinear interpolation algorithm, the eight 2D images were indirectly reconstructed into a 3D image, providing an intuitive visualization of the moisture distribution and the precise location of the ice front, as illustrated in Figure 8.

### 4.4. Finite Element Simulation Verification

**Figure 9:**
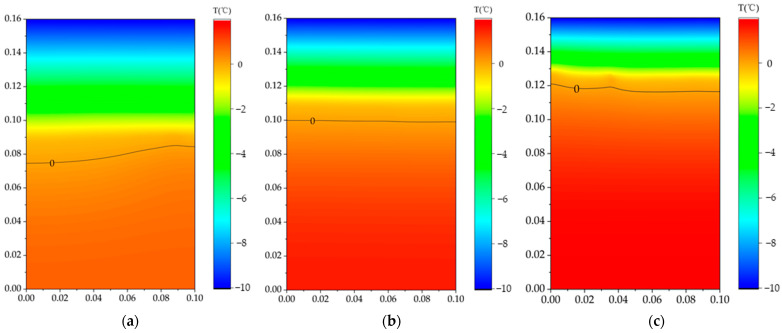
Simulation results of temperature field after 24 h of freezing: (**a**) 10% initial moisture content; (**b**) 15% initial moisture content; (**c**) 20% initial moisture content.

**Figure 10:**
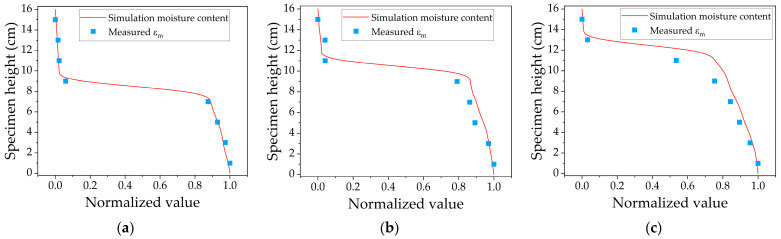
Normalized simulation results of moisture content compared with measured $\varepsilon_{m}$ after 24 h of freezing: (**a**) 10% initial moisture content; (**b**) 15% initial moisture content; (**c**) 20% initial moisture content.

The temperature field distribution of the three models with different initial moisture contents after 24 h is shown in Figure 9. Theoretically, the freezing temperature is 0 °C, but in real conditions, the freezing point may vary due to factors like water salinity and soil composition, typically being below 0 °C. In this finite element simulation, the ice front is defined as the 0 °C surface. The position of the ice front obtained from the $\varepsilon_{m}$ curves is compared with the simulation results, as illustrated in Figure 10.

The simulated unfrozen water content along the depth of the specimen was compared to the $\varepsilon_{m}$ after normalization, with the red solid line representing the model’s solution results and the blue solid dots indicating the results of the laboratory freezing test. The simulation results indicate a downward gradient in moisture content from bottom to top, with the greatest variation near the ice front. Above the ice front, the unfrozen water content approaches zero, and at this point, $\varepsilon_{m}$ value reflects the dielectric properties of the ice–soil mixture. The $\varepsilon_{m}$ index proposed in this paper aligns well with the simulation results, with minimal deviation, as shown in Table 5. This verifies that the $\varepsilon_{m}$ index can accurately represent the moisture distribution in frozen soil and the position of the ice front, whereas $C_{s}$ can only provide a qualitative analysis of moisture distribution without pinpointing the ice front.

## 5. Conclusions

In this study, a self-designed MCCS was employed to evaluate moisture distribution and identify the ice front position in frozen soil. The following conclusions can be summarized from this paper:

1. The fuzzy optimization design method was employed to optimize the structural parameters. Comparing the results with $R_{c}$ and D, using FSI to evaluate the structural parameters of MCCS can simultaneously obtain a low-capacitance dynamic range and a uniformly sensitive field distribution.
2. The sum of the capacitance values ($C_{s}$) and the average dielectric constant distribution ($\varepsilon_{m}$) were introduced to evaluate moisture migration. The point with the minimum slope in the $\varepsilon_{m}$ curve can be considered as the ice front, which was consistent with the moisture content.
3. The trilinear interpolation algorithm was used to reconstruct a 3D image of the ice front. The precise location of the ice front could be identified.
4. The temperature field and unfrozen moisture distribution were obtained via finite element simulation. The $\varepsilon_{m}$ index proposed in this paper aligns well with the simulation results, with minimal deviation.

Future investigations should be implemented to evaluate the accuracy and sensitivity of the method, to develop appropriate reconstruction models and effective numerical methods, and to discover optimal sensor structures and discretization methods for the reconstruction domain for better measurement results.

## Figures and Tables

**Table 1:** Orthogonal experimental design.

Level	A. Electrode Plates Number	B. Electrode Opening Angle Coverage (%)	C. Plate Width (mm)	D. Grounding Shield Radius (mm)
1	8	60	6	58
2	12	70	8	63
3	16	80	10	68

**Table 2:** Optimization indexes results of orthogonal tests.

Experiment	Structural Parameters				Optimization Indexes		
Group	A. Electrode Plates Number	B. Electrode Opening Angle Coverage (%)	C. Plate Width (mm)	D. Grounding Shield Radius (mm)	$R_{c}$	D	FSI
1	8	60	0.6	58	12.453	4.645	0.886
2	8	70	1.0	68	13.282	5.498	0.857
3	8	80	0.8	63	16.907	5.994	0.808
4	12	60	1.0	68	12.758	4.542	0.886
5	12	70	0.8	63	14.089	4.676	0.871
6	12	80	0.6	58	15.330	4.765	0.856
7	16	60	0.8	63	21.668	5.335	0.774
8	16	70	0.6	58	29.150	5.953	0.670
9	16	80	1.0	68	18.876	4.752	0.820

**Table 3:** Range analysis of orthogonal test results.

Level	A. Electrode Plates Number	B. Electrode Opening Angle Coverage	C. Plate Width	D. Grounding Shield Radius
$K_{R1}$	42.176	43.880	53.933	58.815
$K_{R2}$	39.641	56.521	52.664	50.280
$K_{R3}$	69.694	51.112	44.916	42.418
$R_{R}$	30.053	12.641	9.017	16.397
$K_{D1}$	16.137	15.511	15.363	14.073
$K_{D2}$	13.983	16.127	16.005	15.598
$K_{D3}$	16.041	14.523	14.793	16.490
$R_{D}$	2.155	1.604	1.212	2.417
$K_{1}$	2.264	2.568	2.435	2.599
$K_{2}$	2.613	2.397	2.453	2.488
$K_{3}$	2.574	2.486	2.563	2.364
R	0.348	0.171	0.128	0.235

**Table 4:** Optimized parameter results.

Optimal Combination	A_2_B_1_C_3_D_3_	A_2_B_3_C_3_D_1_	A_2_B_1_C_3_D_1_
$R_{c}$	12.758	12.812	12.741
D	4.542	4.537	4.528

**Table 5:** Measurement deviation compared with simulation.

Initial moisture content	10%	15%	20%
Measurement deviation	0.9%	1.5%	2.8%

## Data Availability

Data are contained within the article.
